# Improved outcome of COVID-19 over time in patients treated with CAR T-cell therapy: Update of the European COVID-19 multicenter study on behalf of the European Society for Blood and Marrow Transplantation (EBMT) Infectious Diseases Working Party (IDWP) and the European Hematology Association (EHA) Lymphoma Group

**DOI:** 10.1038/s41375-024-02336-1

**Published:** 2024-07-23

**Authors:** Anne Mea Spanjaart, Per Ljungman, Gloria Tridello, Juana Schwartz, Nuria Martinez-Cibrián, Pere Barba, Mi Kwon, Lucia Lopez-Corral, Joaquin Martinez-Lopez, Christelle Ferra, Roberta Di Blasi, Hervé Ghesquieres, Pim Mutsaers, Friso Calkoen, Margot Jak, Jaap van Doesum, Joost S. P. Vermaat, Marjolein van der Poel, Johan Maertens, Massimiliano Gambella, Elisabetta Metafuni, Fabio Ciceri, Riccardo Saccardi, Emma Nicholson, Eleni Tholouli, Collin Matthew, Victoria Potter, Adrian Bloor, Caroline Besley, Claire Roddie, Keith Wilson, Arnon Nagler, Antonio Campos, Soeren Lykke Petersen, Frantisek Folber, Peter Bader, Jurgen Finke, Nicolaus Kroger, Nina Knelange, Rafael de La Camara, Marie José Kersten, Stephan Mielke

**Affiliations:** 1 Department of Hematology, Amsterdam UMC location University of Amsterdam, Cancer Center Amsterdam and LYMMCARE, Amsterdam, The Netherlands; 2 Department of Cellular Therapy and Allogeneic Stem Cell Transplantation, Karolinska University Hospital Huddinge and Karolinska Comprehensive Cancer Center, Stockholm, Sweden; 3 European Society for Blood and Marrow Transplantation (EBMT) Data Office, Department of Medical Statistics & Bioinformatics, Leiden, Netherlands; 4 European Society for Blood and Marrow Transplantation (EBMT) Leiden Study Unit, European Society for Blood and Marrow Transplantation (EBMT) Data Office, Leiden, Netherlands; 5 Department of Hematology, Hospital Clínic, Barcelona, Spain; 6 Department of Hematology, Vall d’Hebron University Hospital, Barcelona, Spain; 7 Department of Hematology, Hospital G. Universitario Gregorio Marañon, Institute of Health Research Gregorio Marañon, Madrid, Spain; 8 Department of Hematology, Hospital Universitario de Salamanca and IBSAL, Salamanca, Spain; 9 Department of Hematology, Hospital Univ. 12 de Octubre, Complutense University, CNIO, Madrid, Spain; 10 Clinical Hematology Department, Catalan Institute of Oncology, Hospital Germans Trias i Pujol, Josep Carreras Research Institute, Barcelona, Spain; 11 Department of Hematology, Assistance Publique Hôpitaux de Paris—Hopital Saint-Louis, Paris, France; 12 Department of Hematology, Hospices Civils de Lyon, Lyon Sud Hospital, Pierre-Bénite, France; 13 Department of Hematology, Erasmus MC Cancer Center, Rotterdam, the Netherlands; 14 Princess Máxima Center for Pediatric Oncology, Utrecht, the Netherlands; 15 Department of Hematology, University Medical Center Utrecht, Utrecht, Netherlands; 16 Department of Hematology, University Medical Center Groningen, University of Groningen, Groningen, Netherlands; 17 Department of Hematology, Leiden University Medical Center, Leiden, Netherlands; 18 Department of Hematology, Department of Internal Medicine, Division of Hematology, GROW School for Oncology and Developmental Biology, Maastricht University Medical Center, Maastricht, Netherlands; 19 Deptartment of Hematology, University Hospital Gasthuisberg, Leuven, Belgium; 20 Department of Hematology and Cellular Therapy, IRCCS Ospedale Policlinico San Martino, Genova, Italy; 21 Dipartimento di Diagnostica per Immagini, Radioterapia Oncologica e Ematologia, Fondazione Policlinico Universitario Agostino Gemelli IRCCS, Rome, Italy; 22 Hematology and BMT Unit IRCCS San Raffaele Scientific Institute, Milan, Italy; 23 Cell Therapy and Transfusion Medicine Unit Azienda Ospedaliero Universitaria Careggi, Firenze, Italy; 24 Department of Haematology, The Royal Marsden Hospital, London, United Kingdom; 25 Department of Clinical Haematology, Manchester Royal Infirmary, Manchester University NHS Foundation Trust, Manchester, UK; 26 Adult HSCT unit, Northern Centre for Bone Marrow Transplantation, Newcastle Tyne, UK; 27 King’s College Hospital NHS Foundation Trust, Department of Haematological Medicine, Denmark Hill, London, UK; 28 Adult Leukaemia and Bone Marrow Transplant Unit, Christie NHS Foundation Trust Hospital, University of Manchester, Manchester, UK; 29 Department of Haematology, University Hospitals Bristol and Weston NHSFT, Bristol, UK; 30 Department of Haematology, University College London Hospital, London, UK; 31 Blood and Bone Marrow Transplantation Department, University Hospital of Cardiff, Cardiff, UK; 32 Chaim Sheba Medical Center, Tel Aviv University, Tel Hashomer, Israel; 33 Celular Therapy Department, Instituto Portugués de Oncologia do Porto, Francisco Gentil, E.P.E, Porto, Portugal; 34 Department of Hematology, Copenhagen University Hospital, Rigshospitalet, Copenhagen, Denmark; 35 Department of internal Medicine, Hematology and Oncology, Masaryk University Hospital Brno, Brno, Czech Republic; 36 Department for Children and Adolescents, Division for Stem Cell Transplantation and Immunology, University Hospital Frankfurt, Goethe University, Frankfurt, Germany; 37 Department of Hematology/Oncology/Stem Cell Transplantation, Faculty of Medicine and Medical Center, University of Freiburg, Freiburg im Breisgau, Germany; 38 Department of Stem cell Transplantation, University Hospital Eppendorf, Hamburg, Germany; 39 Department of Hematology, Hospital Universitario de La Princesa, Madrid, Spain; 40 Department of Cellular Therapy and Allogeneic Stem Cell Transplantation (CAST), Department of Laboratory Medicine, Karolinska Institutet and University Hospital, Karolinska Comprehensive Cancer Center, Karolinska ATMP Center, Stockholm, Sweden; 41 Cellular Therapy and immunobiology working party (CTIWP) of EBMT, https://www.ebmt.org/working-parties/cellular-therapy-immunobiology-working-party-ctiwp

**Keywords:** Immunotherapy, Haematological cancer

## Abstract

COVID-19 has been associated with high mortality in patients treated with Chimeric Antigen Receptor (CAR) T-cell therapy for hematologic malignancies. Here, we investigated whether the outcome has improved over time with the primary objective of assessing COVID-19-attributable mortality in the Omicron period of 2022 compared to previous years. Data for this multicenter study were collected using the MED-A and COVID-19 report forms developed by the EBMT. One-hundred-eighty patients were included in the analysis, 39 diagnosed in 2020, 35 in 2021 and 106 in 2022. The median age was 58.9 years (min-max: 5.2–78.4). There was a successive decrease in COVID-19-related mortality over time (2020: 43.6%, 2021: 22.9%, 2022: 7.5%) and in multivariate analysis year of infection was the strongest predictor of survival (*p* = 0.0001). Comparing 2022 with 2020–2021, significantly fewer patients had lower respiratory symptoms (21.7% vs 37.8%, *p* = 0.01), needed oxygen support (25.5% vs 43.2%, *p* = 0.01), or were admitted to ICU (5.7% vs 33.8%, *p* = 0.0001). Although COVID-19-related mortality has decreased over time, CAR T-cell recipients remain at higher risk for complications than the general population. Consequently, vigilant monitoring for COVID-19 in patients undergoing B-cell-targeting CAR T-cell treatment is continuously recommended ensuring optimal prevention of infection and advanced state-of-the art treatment when needed.

## Introduction

It was previously reported that patients with hematologic malignancies treated with B-cell-directed Chimeric Antigen Receptor (CAR) T-cell therapy are facing a substantially increased morbidity and mortality following an infection with severe adult respiratory syndrome coronavirus 2 (SARS‐CoV‐2) causing coronavirus disease 2019 (COVID-19) when compared to the general population [1, 2]. Factors considered to be associated with a higher mortality risk were ongoing B-cell aplasia, T-cell depletion, hypogammaglobulinemia, and cytopenias; all leading to a severely immunocompromised state. During the pre-Omicron period, when only a minority of CAR T-cell recipients were vaccinated, we, as the European Society for Blood and Marrow Transplantation Infectious Disease Working Party (EBMT IDWP) and European Hematology Association (EHA) Lymphoma Group reported a COVID-19-attributable mortality rate of 41% [1]. Since then, much has changed as patients have been offered multiple vaccine doses and new therapies have become available. In addition, many patients probably have had prior SARS-CoV-2 infections and as has been shown in several studies, hybrid immunity most likely confers a lower risk for severe COVID-19 [3, 4]. Furthermore, SARS-CoV-2 variants less prone to cause severe lower respiratory tract disease have emerged [5, 6]. In an analysis from the EBMT registry of allogeneic hematopoietic stem cell transplantation recipients (allo-HCT), a major improvement in outcome was shown. The mortality during the early Omicron period in 2022 was only 4.5% compared to almost 25% during the first phase of the pandemic. Moreover, the mortality in vaccinated allo-HCT patients was only 1% [7]. On the other hand CAR T-cell recipients have shown substantially impaired humoral responses to vaccination whilst the exact role of cellular mediated immunity remains uncertain [8–11]. Given this decreased vaccine immunogenicity, additional therapeutic interventions are strongly recommended [12, 13]. The aim of this study was to analyze whether the outcome of SARS-CoV-2 infection in CAR T-cell recipients has improved over time, particularly between March 2020 and December 2022, information very much needed for optimized clinical decision making based on appropriate risk stratification.

## Subjects and methods

The COVID-19 report form developed by the EBMT was updated and used for this multicenter survey study (www.ebmt.org). All patients gave informed consent to have their data reported to the EBMT registry. The Swedish central Ethical Board (EPM 2020-01731, 2021-04692) approved the study and other approvals, if required, were obtained according to national regulations.

CAR T-cell recipients treated for hematological B-cell malignancies with either a positive SARS-CoV-2 PCR or antigen-test diagnosed before January 2023 and at least six weeks of follow-up after initial SARS-CoV-2 diagnosis (unless the patient had died) were included. Data on baseline clinical characteristics, COVID-19 infection, management, and outcome were collected. Patients were split in three calendar years (2020, 2021, 2022). The period since the beginning of the pandemic until June 2021 includes patients previously reported [1]. Patients were considered fully vaccinated after receiving at least 3 doses of any COVID-19 vaccine. Virologic resolution of SARS-CoV-2 infection was defined as the time from the first positive PCR or antigen test until the first negative PCR test. Clinical resolution was defined as the time from the first positive PCR or antigen test until the first day when no clinical COVID-19-related signs were present. Metabolic comorbidity was defined as the presence of one or more of the following conditions: obesity, hypertension, diabetes, hypercholesterolemia, active smoking, and cardiovascular disease. The primary objective of this study was to assess the COVID-19-attributable mortality in the Omicron period of 2022 and compare it to previous years. Secondary objectives were to evaluate the frequencies of lower respiratory tract symptoms, oxygen support, hospital- and ICU admission and to explore factors associated with mortality.

## Statistics

Descriptive statistics were used for clinical characteristics. For continuous variables the median, minimum and maximum values were used and for categorical variables the absolute and percentage frequencies. The continuous variables between groups were compared using the Kruskal-Wallis or one-way-ANOVA test and the categorical variables between groups were compared using the Chi-Square or Fisher-exact test (as appropriate). The overall survival (OS) was estimated using the Kaplan-Meier method, considering death due to any cause as an event and time from SARS-CoV-2 infection to the last date of follow-up as survival time. Risk factors were evaluated using a Cox proportional hazard model. We applied multiple significance testing in several 3-factor multivariable models due to the limited number of events. A p-value < 0.05 was considered statistically significant. All p-values are two-sided. The statistical software SAS v. 9.4 (SAS Institute Inc., Cary, NC, USA) was used to perform the main analyses.

## Results

### Patients

One hundred eighty patients from 12 different countries were included. Thirty-nine patients were reported in 2020, 35 in 2021 and 106 in 2022. Table 1 displays demographic and clinical characteristics. The median age was 58.9 years (min-max: 5.2–78.4), including 178 adults and 2 children (<18-years old). Patients in 2022 were older (median 61.3, min-max: 20.2–78.4) than in 2020–2021 (median 56.2, min-max: 5.2–72.8). Sixty-eight patients were female (37.8%). The majority of patients received CAR T-cell therapy for B-cell-non-Hodgkin lymphoma (82.2%). Most patients were in complete remission (CR) at time of SARS-CoV-2 infection (72.2%) and this proportion did not significantly change between 2020–2021 and 2022. Additional characteristics (e.g. type of CAR T-cell therapy, prior allo-HCT, IVIG substitution for hypogammaglobulinemia) can be found in Supplementary Table 1.

**Table 1:** Baseline demographics and clinical characteristics of patients infected by SARS-CoV-2 per year of inclusion and differences over the years.

Characteristics	Year			Total	*P*-value
	2020	2021	2022		Δ 2020–2021 versus 2022^#^
	*N* = 39	*N* = 35	*N* = 106	*N* = 180	
Age, median age in years (min-max) at COVID-19	58.5 (5.2–72.8)	55.2 (17.6–72.6)	61.3 (20.2–78.4)	58.9 (5.2–78.4)	
	56.2 (5.2–72.8)		61.3 (20.2–78.4)		0.01**
Female sex, N (%)	17 (43.6)	17 (48.6)	34 (32.1)	68 (37.8)	0.06
CAR T-cell therapy indication, N (%)					0.7
Acute leukemia	5 (12.8)	5 (14.3)	10 (9.4)	20 (11.1)	
B-NHL	32 (82.1)	27 (77.1)	89 (84.0)	148 (82.2)	
Multiple myeloma	2 (5.1)	3 (8.6)	7 (6.6)	12 (6.7)	
Disease status at time of COVID-19 diagnosis, N (%)					0.09
Relapse/Progression	8 (20.5)	7 (20)	13 (12.3)	28 (15.5)	
Partial response	4 (10.3)	5 (14.3)	9 (8.5)	18 (10)	
Complete response	27 (69.2)	22 (62.9)	81 (76.4)	130 (72.2)	
Unknown	0 (0.0)	1 (2.9)	3 (2.8)	4 (2.2)	
Metabolic comorbidity*, N (%)	11 (28.2)	10 (28.6)	36 (34.0)	57 (31.7)	0.3
Unknown	0 (0.0)	5 (14.3)	10 (9.4)	15 (8.3)	
Lansky/Karnofsky-score < 70 at COVID-19 diagnosis, N (%)	9 (23.1)	3 (8.6)	8 (7.5)	20 (11.1)	0.1
Unknown	0 (0.0)	1 (2.9)	15 (14.2)	16 (8.9)	
Median time from CAR-T to COVID-19 diagnosis, months (min days-max months)	7.7 (1–17.5)	7.4 (6–37.1)	6.4 (5–42.5)	7.2 (1–42.5)	0.6
Symptoms at time of COVID-19 diagnosis, N (%)					
Asymptomatic	5 (12.8)	3 (9.1)	14 (13.5)	22 (12.2)	0.6
Fever	26 (66.7)	19 (54.3)	46 (43.4)	91 (50.6)	
	45 (60.8)		46 (43.4)		0.02**
Upper respiratory symptoms	17 (43.6)	13 (37.1)	64 (60.4)	94 (52.2)	
	30 (40.5)		64 (60.4)		0.02**
Lower Respiratory symptoms	15 (38.5)	13 (37.1)	23 (21.7)	51 (28.3)	
	28 (37.8)		23 (21.7)		0.01**
Oxygen support, N (%)	18 (46.2)	14 (40.0)	27 (25.5)	59 (32.8)	
	32 (43.2)		27 (25.5)		0.01**
Missing	0 (0.0)	3 (8.6)	3 (2.8)	6 (3.3)	
Invasive mechanical ventilation	8/18 (44.4)	7/14 (50.0)	3/27 (11.1)	18/59 (30.5)	
Hospital admission, N (%)	32 (82.1)	23 (65.7)	46 (43.4)	101 (56.1)	
	55 (74.3)		46 (43.4)		<0.0001**
Missing	0 (0.0)	2 (5.7)	0 (0.0)	2 (1.1)	
Median hospital admission duration, days (min-max)	23.5 (1–93)	18 (2–82)	11 (1–280)	17.5 (1–280)	
	21(1–93)		11 (1–280)		
ICU admission, N (%)	15 (38.5)	10 (28.6)	6 (5.7)	31 (17.2)	
	25 (33.8)		6 (5.7)		<0.0001**
Missing	1 (2.6)	1 (2.9)	0 (0.0)	2 (1.1)	
Median ICU admission duration, days (min-max)	20.0 (2–68)	17.5 (7–38)	7.5 (3–57)	13.5 (2–68)	
	19 (2–68)		7.5 (3–57)		

*B-NHL* B-cell Non-Hodgkin Lymphoma.

^*^Metabolic comorbidities include obesity, hypertension, diabetes, hypercholesterolemia, smoking and cardiovascular disease ** significant. ^#^ The *P* values given in this table are for the comparison of the years 2020 and 2021 combined with the year 2022 ** significant *P* value.

### Previous vaccination

Seventy-four of 180 patients were reported to have received at least one vaccine dose prior to the SARS-CoV-2 diagnosis (41.1%). Forty-seven of these patients (26.1%) received at least 3 vaccine doses and were considered fully vaccinated. Twenty of the 74 vaccinated patients had received at least 2 vaccine doses before their CAR T-cell infusion. The percentage of fully vaccinated patients significantly increased in 2022 compared to 2021 (no vaccine available in 2020; Table 2).

**Table 2:** COVID-19 vaccination status prior to SARS-CoV-2 diagnosis.

	2020 (*N* = 39)	2021 (*N* = 35)	2022 (*N* = 106)	Total (*N* = 180)	*P* value Δ 2021 versus 2022
COVID-19 vaccination status					
No vaccination	39 (100.0)	21 (60.0)	15 (14.2)	75 (41.7)	
1–2 vaccinations	0 (0.0)	7 (20.0)	20 (18.9)	27 (15.0)	
≥3 vaccinations	0 (0.0)	1 (2.9)	46 (43.4)	47 (26.1)	<0.0001
Unknown	0 (0.0)	6 (17.1)	25 (23.6)	31 (17.2)	

### SARS-CoV-2 infection

The median time from CAR-T-cell infusion to SARS-CoV-2 infection was 7.2 months (min-max: 1 day - 42.5 months) and did not significantly differ between 2020–2021 and 2022. 12.2% of the patients were asymptomatic and this proportion of patients did not significantly differ between 2020–2021 and 2022. At the time of diagnosis, fewer patients had lower respiratory tract symptoms in 2022 compared to 2020 and 2021 (2020–2021: 37.8%, 2022: 21.7%, *p* = 0.01) and fever (2020–2021: 60.8%, 2022: 43.4%, *p* = 0.02). Conversely, more patients had upper respiratory symptoms in 2022 compared to 2020 and 2021 (2020–2021: 40.5%, 2022; 60.4%, *p* = 0.02). The proportion of patients hospitalized during a COVID-19 episode decreased over time (2020–2021: 74.3%, 2022: 43.4%, *p* < 0.0001) as did the proportion of patients needing admission to the ICU (2020–2021: 33.8%, 2022: 5.7%, *p* < 0.0001). In 2022, the duration of hospital admission was shorter (11 days versus 21 days) as was the time in an ICU (19 days versus 7.5 days). Furthermore, fewer patients needed oxygen support (2020–2021: 43.2%, 2022: 25.5%, *p* = 0.01). Available laboratory values at time of SARS-CoV-2 diagnosis can be found in Supplementary Table 2.

### COVID-19 treatment

The proportion of patients treated with monoclonal antibodies (Moabs) increased over time (0% in 2020, 8.6% in 2021 and 14.2% in 2022) and 3.8% of patients had received pre-exposure tixagevimab/cilgavimab in 2022. The proportion of patients receiving antiviral drugs remained stable over time (28.2% in 2020, 31.4% in 2021 and 34.6% in 2022). However, the drugs given changed over time (11.1% of the patients received nirmatrelvir/ritonavir in 2022 compared with none during 2020 and 2021; Table 3 and Supplementary Table 3).

**Table 3:** COVID-19 drugs used for treatment.

	2020 (*N* = 39)	2021 (*N* = 35)	2022 (*N* = 106)	Total (*N* = 180)
Drug				
Corticosteroids	8 (20.5)	7 (20)	20 (18.9)	35 (19.4)
Anti-inflammatory drugs*	6 (15.4)	6 (17.1)	11 (10.4)	23 (12.8)
Antiviral therapy				
Remdesivir	11 (28.2)	11 (31.4)	24 (22.6)	46 (25.6)
Molnupiravir	0 (0.0)	0 (0.0)	1 (0.9)	1 (0.6)
Nirmatrelvir/Ritonavir	0 (0.0)	0 (0.0)	20 (11.1)	20 (11.1)
Monoclonal antibodies	0 (0.0)	3 (8.6)	15 (14.2)	18 (10)
Convalescent plasma	9 (23.1)	8 (22.9)	12 (11.3)	29 (16.1)
Pre-exposure monoclonal antibodies	0 (0.0)	0 (0.0)	4 (3.8)	4 (2.2)

*The given anti-inflammatory drugs were Tocilizumab, Siltuximab, Anakinra, Baricitinib and Eculizumab and no use of Ruxolitinib, Sarilumab or Colchicine was reported.

### Mortality

**Fig. 1:**
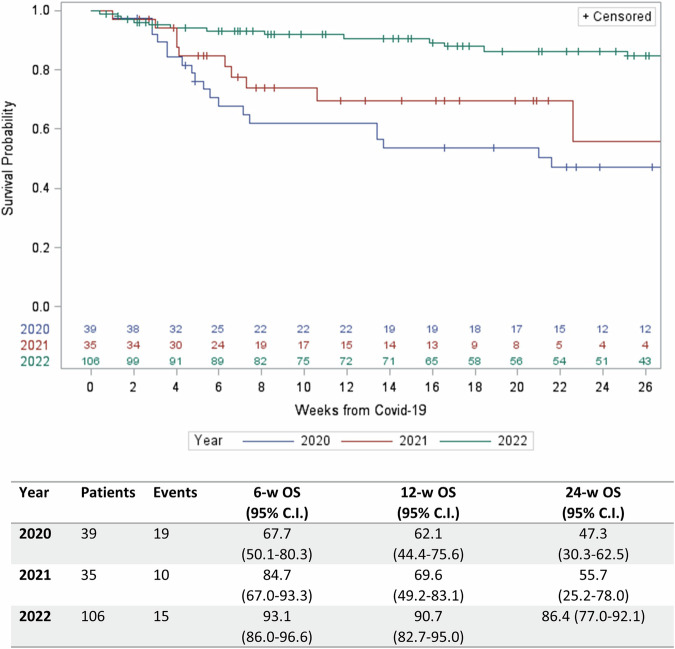
Overall survival of patients after COVID-19 diagnosis per year. Overall survival was estimated using the Kaplan-Meier method, considering death due to any cause as an event and time from COVID-19 infection to the last date of follow-up as survival time.

At the time of analysis 44 of 180 patients had died (24.4%) of whom 33 (75%) died from COVID-19. The COVID-19-attributable mortality decreased in 2022 to 7.5% (2020: 43.6% and 2021: 22.9%; Table 4). OS per year is shown in Fig. 1. Three of 47 fully vaccinated patients, died due to COVID-19 (6.4%). Risk factors associated with mortality in univariate analysis were older age (10 year effect, HR 1.46, 95% CI 1.14–1.86, *p* = 0.003), having a metabolic comorbidity (HR 2.53, 95% CI 1.39–4.62, *p* = 0.003), shorter time between CAR T-cell infusion and SARS-CoV-2 infection (≤3 months, HR 2.41, 95% CI 1.29–4.51, *p* = 0.006), remission status (no CR, HR 4.99, 95% CI 2.74–9.09, *p* < 0.0001), platelet count (>75 × 10^9^/L, HR 0.32, 95% CI 0.17–0.61, *p* < 0.001), performance status, (10 points effect, HR 0.68, 95% CI 0.60-0.77, *p* < 0.0001), not being fully vaccinated before SARS-CoV-2 infection (HR 5.64, 95% CI 1.72-18.47, *p* = 0.004) and diagnosis of SARS-CoV-2 infection in the years 2020–2021 (year 2020, HR 3.92, 95% CI 1.99-7.74, year 2021, HR 2.65, 95% CI 1.18-5.93, year 2020–2021, HR 3.37, 95% CI 1.80–6.31, *p* = 0.0001; Supplementary Table 4). For the period with the highest mortality, the first 3 months after CAR T-cell infusion, mortality decreased in 2021 and 2022, (2020): 86% (6/7); 2021: 20% (1/5); 2022: 31% (8/26). Several 3-factor multivariate models were constructed testing for age, performance status, remission status, year of SARS-CoV-2 infection and time between CAR T-cell infusion and SARS-CoV-2 infection. In these models, all factors remained significant (Supplementary Table 5).

**Table 4:** COVID-19-attributable mortality and resolution of COVID-19.

	2020 (*N* = 39)	2021 (*N* = 35)	2022 (*N* = 106)	Total (*N* = 180)
Non-COVID-19 related mortality, N (%)	2 (5.1)	2 (5.7)	7 (6.6)	11 (6.1)
COVID-19 related mortality, N (%)	17 (43.6)	8 (22.9)	8 (7.5)	33 (18.3)
Median time between COVID-19 until death, days (min-max)	37 (7–151)	37 (7–158)	57 (3–283)	39 (3–283)
Median follow-up time from SARS-CoV-2 diagnosis, days (min-max)	96 (7–425)	60 (7–332)	158 (3–343)	119 (3–425)
Virologic (PCR) resolution of SARS-CoV-2 in surviving patients, N (%)	14 (70.0)	15 (60.0)	47 (51.6)	76 (55.9)
Median duration virologic SARS-CoV-2 infection, days (min-max)	24 (6–94)	54 (13–332)	51 (5–221)	50 (5–332)
Clinical resolution COVID-19 in surviving patients, N (%)	6 (30.0)	9 (36.0)	38 (41.8)	53 (39.0)
Median duration clinical COVID-19, days (min-max)	31 (7–157)	13 (4-82)	22 (5–218)	21 (4–218)
Missing data COVID-19 resolution, N (%)	0 (0.0)	1 (2.9)	6 (5.7)	7 (3.9)

### Resolution

For patients surviving SARS-CoV-2 infection, the median time to clinical resolution was 21 days (min–max 4-218) and the median time to virologic (PCR) resolution 50 days (min–max 5-332; Table 4).

## Discussion

The emergence of the COVID-19 pandemic had a tremendous impact on CAR T-cell therapy both in clinical trials and in standard of care. Previous reports, describing the pre-Omicron and the pre-vaccination period, showed clearly that these patients were one of the most vulnerable groups in the population with high risks for severe and prolonged disease and death in up to 50% of patients [1, 2]. Since these early reports, there have been several important developments. These include the introduction of population-based vaccination, introduction of new COVID-19 therapies, and the emergence of new SARS-CoV-2 variants, possibly all contributing to the lower mortality observed in allo-HCT- and lymphoid malignancy patients (4.5–7%) [7, 8, 14]. A cohort study of 64 CAR T-cell recipients that looked at the impact of COVID-19 vaccination and Moab use, reported a COVID-19-attributable mortality of 13% in 2022 [15]. In a retrospective analysis of 75 children and young adults receiving CAR T-cell therapy the mortality rate was 4.3%. The admission rate for SARS-CoV-2 infections was nearly 10 times higher in the pre-Omicron period (40.4%) compared to the Omicron period (4.3%), with 95.7% of patients having asymptomatic or mild SARS-CoV-2 infection after the emergence of the Omicron variant [16].

In this study, describing the largest cohort of CAR T-cell recipients with SARS-CoV-2 infection to date, we report that the COVID-19-related mortality was significantly reduced over time, with a COVID-19-attributable mortality rate of 43.6% at the beginning of the pandemic that had decreased to 7.5% in 2022 (the Omicron period). Furthermore, significantly fewer patients had lower respiratory symptoms, needed oxygen support, or had to be admitted to the hospital or ICU, reflecting a much lower severity of COVID-19. Increasing age has been one of the most important factors associated with worse outcome of COVID-19 in the general population [17]. Restricted by a limited number of events, we explored whether the year of SARS-CoV-2 infection and time from CAR T-cell therapy were associated with OS in different 3-factor multivariate models together with previously identified factors such as age, performance status and tumor remission status [1]. All these factors had significant impact on OS. The impact of SARS-COV-2 infection occurring early after CAR T cell therapy is similar to the finding in allo-HCT recipients [7]. As the Omicron period coincided with more patients being (fully) vaccinated and the availability of new therapeutic modalities, it is difficult to disentangle the relative contribution of the Omicron variant, previous SARS-CoV-2 infections, vaccinations, and new COVID-19 therapies. In the general population, newer SARS-CoV-2 variants have been associated with progressively less lung involvement and decreasing mortality, even in unvaccinated patients [18]. This has been particularly shown comparing the Omicron with the Delta variants [19]. Although vaccination is significantly associated with better survival after SARS-CoV-2 infection, vaccine effectiveness can’t be assessed in this study since we only assessed breakthrough infections.

Patients with ongoing B-cell aplasia such as patients treated with CAR T-cell therapy still have a diminished humoral response to COVID-19 vaccination with multiple dose regimens [10, 20]. These findings are in line with patients with B-cell aplasia due to primary immunodeficiency syndromes, such as XLP. However, from a large study in immunocompromised patients including patients with inborn B-cell defects it became evident that these patients have the principle capacity to mount significant T-cell responses against COVID-19 [21]. Robust cellular responses can also be detected in the majority of CAR T-cell recipients and proportions of spike-specific CD8 + T-cells even seem to be significantly higher in the absence of a humoral response [22, 23]. Although, CD8 + T-cells are associated with improved survival in B-cell depleted patients with hematologic malignancies and COVID-19, the true protective value of T-cell responses remains uncertain [24]. The finding that patients have an increased risk of dying during the first 3 months after CAR T-cell therapy can possibly be explained by the fact that patients in this period do not only have B-cell aplasia, but are also severely T-cell depleted due to the lymphodepleting chemotherapy preceding CAR T-cell infusion. The recommendations regarding timing of vaccination vary. Some groups recommend starting as early as 3 months after CAR T-cell infusion, while others recommend individual consideration based on the immune status of the patient [12, 25]. Furthermore, revaccination is advised given the apprehension about potential immunity loss after CAR T-cell infusion. However, it is important to note that the optimal revaccination regimen remains to be determined in studies [9, 26]. The potentially very long time to virologic COVID-19 resolution clearly shows that prolonged viral shedding remains a problem in CAR T-cell recipients. B-cell aplasia with diminished neutralizing antibodies seems to be the main risk factor [27]. The optimal duration and isolation measures for infection prevention are still undetermined due to the absence of standardized tests capable of differentiating between infectious virus and non-viable RNA [28]. The combination of convalescent plasma with remdesivir, as well as antiviral combinations, have shown possible effects on SARS-CoV-2 clearance in immunocompromised patients [29–31].

Limitations of this study is its retrospective nature, difficulties to ensure that all patients especially those with mild infections were reported, the lack of information about the specific SARS-CoV-2 variants infecting the patients, and missing data regarding B- and T-cell recovery after CAR T-cell therapy at time of SARS-CoV-2 diagnosis.

We conclude that COVID-19 related morbidity and mortality has been significantly reduced over time. Nonetheless CAR T-cell recipients remain at much higher risk than the general population. Therefore, the vulnerability of patients after B-cell directed CAR T-cell therapy warrants further careful monitoring together with access to and the development of preventive measures and COVID-19 treatments, including vaccinations, antivirals and monoclonal antibodies. In addition, the potential effects of COVID-19 infections on off-target side-effects associated with CAR T-cell therapy such as cytokine-release-syndrome (CRS), neurotoxicity (ICANS), hematological toxicity (ICAHT) and in particular macrophage-activation syndrome (MAS) are poorly understood and need to be studied much more extensively.

### Supplementary information

Supplementary data file
