# Supplementary material for: Improved outcome of COVID-19 over time in patients treated with CAR T-cell therapy: Update of the European COVID-19 multicenter study on behalf of the European Society for Blood and Marrow Transplantation (EBMT) Infectious Diseases Working Party (IDWP) and the European Hematology Association (EHA) Lymphoma Group
Source: Leukemia. 2024 Jul 23;38(9):1985–91. doi: 10.1038/s41375-024-02336-1 (PMC11347385; doi:10.1038/s41375-024-02336-1)
Supplement: Supplementary file 1 — Supplementary data file [file 41375_2024_2336_MOESM1_ESM.docx]

**SUPPLEMENTARY DATA**

Improved outcome of COVID-19 over time in patients treated with CAR T-cell therapy: update of the European COVID-19 multicenter study on behalf of the European Society for Blood and Marrow Transplantation (EBMT) Infectious Diseases Working Party and the European Hematology Association (EHA) Lymphoma Group

**Supplementary Table 1**. Clinical characteristics of patients with SARS-CoV-2 infection

| **Characteristics** | **Year** | | | **Total** |
| --- | --- | --- | --- | --- |
|  | **2020 (N=39)** | **2021 (N=35)** | **2022 (N=106)** | **N=180** |
| Prior allo-HCT  Missing information | 3 (7.7)  1 (2.6) | 5 (14.3)  2 (5.7) | 7 (6.6)  3 (2.8) | 15 (8.3)  6 (3.3) |
| Lung disease before COVID-19  Missing information | 8 (20.5)  6 (15.4) | 1 (2.9)  6 (17.1) | 9 (8.5)  2 (1.9) | 18 (10.0)  14 (7.8) |
| CD28  41BB  CD28 & 41BB  Missing information | 15 (38.5)  19 (48.7)  2 (5.1)  3 (7.7) | 18 (51.4)  14 (40.0)  1 (2.9)  2 (5.7) | 57 (53.8)  38 (35.8)  0 (0.0)  11 (10.4) | 90 (50.0)  71 (39.4)  3 (1.7)  16 (8.9) |
| No CRS  CRS ≥ grade 3  Missing information | 12 (30.8)  1 (2.6)  3 (7.7) | 5 (14.3)  3 (8.6)  2 (5.7) | 11 (10.4)  5 (4.7)  3 (2.8) | 28 (15.6)  9 (5.0)  8 (4.4) |
| No ICANS  ICANS ≥ grade 3  Missing information | 32 (82.1)  1 (2.6)  3 (7.7) | 24 (68.6)  3 (8.6)  2 (5.7) | 60 (56.6)  19 (17.9)  3 (2.8) | 116 (64.4)  23 (12.8)  8 (4.4) |
| IVIG substitution for hypogammaglobulinemia after CAR T-cell therapy  No IVIG substitution  Missing information | 0 (0.0)  0  39 (100.0) | 1 (2.9)  1 (2.9)  33 (94.3) | 18 (17.0)  72 (67.9)  16 (15.1) | 19 (10.6)  73 (40.6)  88 (48.9) |

Abbreviations; allo-HCT: allogeneic stem cell transplant, CRS: cytokine release syndrome, ICANS: immune effector cell-associated neurotoxicity syndrome, IVIG: intravenous immunoglobulin

**Supplementary Table 2**. Laboratory values at time of SARS-CoV-2 diagnosis

| **Characteristics** | **Year** | | | **Total** |
| --- | --- | --- | --- | --- |
|  | **2020 (N=39)** | **2021 (N=35)** | **2022 (N=106)** | **N=180** |
| Neutrophils (x10 9 /L)  N | 2.3, 0.0 - 19.9  35 | 1.7, 0.2 - 5.2  25 | 1.3, 0.0 - 8.1  72 | 1.6, 0.0 - 19.9  132 |
| Lymphocytes (x10 9 /L)  N | 0.6, 0.0 - 6.9  34 | 0.5, 0.1 - 9.0  23 | 0.7, 0.0 - 5.0  68 | 0.6, 0.0 - 9.0  125 |
| IgG (g/l)  N | 3.0, 0.4 - 18.0  21 | 4.3, 1.0 - 9.8  13 | 3.3, 0.6 - 6.7  29 | 3.4, 0.4 - 9.8  63 |
| Platelets (x10 9/L)  N | 116.0, 15.5 - 481.0  37 | 112.0, 8.0 - 399.0  25 | 106.0, 14.0 - 335.0  75 | 109.0, 8.0 - 481.0  137 |
| CRP (mg/L)  N | 27.0, 0.2 - 264.0  31 | 25.0, 0.4 - 253.4  19 | 15.0, 0.0 - 264.0  58 | 19.8, 0.0 - 264.0  108 |

**Supplementary Table 3.** Types of anti-inflammatory drugs given as COVID-19 treatment

| **Anti-inflammatory drug** | **2020 (N=39)** | **2021(N=35)** | **2022 (N=106)** | **Total (N=180)** |
| --- | --- | --- | --- | --- |
| Tocilizumab | 5 (12.8) | 4 (11.4) | 8 (7.6) | 17 (9.4) |
| Siltuximab | 0 (0.0) | 0 (0.0) | 1 (0.9) | 1 (0.56 |
| Anakinra | 0 (0.0) | 0 (0.0) | 2 (1.9) | 2 (1.1) |
| Baricitinib | 0 (0.0) | 2 (5.7) | 0 (0.0) | 2 (1.1) |
| Eculizimab | 1 (2.6) | 0 (0.0) | 0 (0.0) | 1 (0.6) |

**Supplementary Table 4.** Risk factors associated with mortality in univariate analysis

| **Variable** |  | **Patients** | **Deaths** | **Univariate analysis** | |
| --- | --- | --- | --- | --- | --- |
|  |  |  |  | HR (95% C.I.) | P |
| Age | | | | | |
|  | Continuous  10 year effect | 180 | 44 | 1.46 (1.14-1.86) | 0.003 |
| Sex | | | | | |
|  | Male | 112 | 32 | 1,00 |  |
|  | Female | 68 | 12 | 0.54 (0.28-1.04) | 0.065 |
| Time from CAR T-cell infusion to COVID-19 | | | | | |
|  | Continuous | 180 | 44 | 0.93 (0.89-0.98) | 0.005 |
|  | ≤ 3 months | 38 | 15 | 2.41 (1.29-4.51) | 0.006 |
|  | > 3 months | 142 | 29 | 1.00 |  |
| Metabolic comorbidity | | | | | |
|  | Yes | 57 | 24 | 2.53 (1.39-4.62) | 0.003 |
|  | No | 108 | 19 | 1.00 |  |
| Pre-existing lung pathology | | | | | |
|  | Yes | 18 | 6 | 1.74 (0.73-4.16) | 0.21 |
|  | No | 148 | 33 | 1.00 |  |
| CRS ≥ grade 2 | | | | | |
|  | Yes | 58 | 15 | 1.04 (0.55-1.95) | 0.9 |
|  | No | 114 | 27 | 1.00 |  |
| ICANS ≥ grade 2 | | | | | |
|  | Yes | 38 | 7 | 0.73 (0.32-1.64) | 0.4 |
|  | No | 134 | 36 | 1.00 |  |
| Vaccination status fully vaccinated before COVID-19 | | | | | |
|  | Yes | 47 | 3 | 1.00 |  |
|  | No | 102 | 31 | 5.64 (1.72-18.47) | 0.004 |
| Tumor remission status at time of COVID | | | | | |
|  | CR | 130 | 19 | 1.00 |  |
|  | No CR | 46 | 25 | 4.99 (2.74-9.09) | <0.0001 |
| Performance status | | | | | |
|  | 10 point effect | 164 | 40 | 0.68 (0.60-0.77) | <0.0001 |
| Year of SARS-CoV-2 infection diagnosis | | | | | |
|  | 2020 | 39 | 19 | 3.92 (1.99-7.74) | <0.0001 |
|  | 2021 | 35 | 10 | 2.65 (1.18-5.93) | 0.02 |
|  | 2022 | 106 | 15 | 1.00 | 0.0004 |
| Year of SARS-CoV-2 infection diagnosis | | | | | |
|  | 2020-2021 | 74 | 29 | 3.37 (1.80-6.31) | 0.0001 |
|  | 2022 | 106 | 15 | 1.00 |  |
| Neutrophils at time of screening for SARS-CoV-2/diagnosis | | | | | |
|  | ≤ 0.5 | 13 | 4 | 1.00 |  |
|  | > 0.5 | 119 | 29 | 0.74 (0.26-2.10) | 0.6 |
| Lymphocytes at time of screening for SARS-CoV-2/diagnosis | | | | | |
|  | ≤ 1 | 89 | 20 | 1.00 |  |
|  | >1 | 37 | 10 | 1.36 (0.64-2.92) | 0.4 |
| Platelets at time of screening for SARS-CoV-2/diagnosis | | | | | |
|  | ≤ 75 | 43 | 19 | 1.00 |  |
|  | >75 | 94 | 18 | 0.32 (0.17-0.61) | 0.0006 |
| IgG at time of screening for SARS-CoV-2/diagnosis | | | | | |
|  | ≤ 4 | 34 | 9 | 1.00 |  |
|  | > 4 | 33 | 5 | 0.52 (0.17-1.55) | 0.24 |

**Supplementary Table 5.** Multivariate analysis for mortality

| **Variable** |  | **Patients** | **Deaths** | **Multivariate analysis** | |
| --- | --- | --- | --- | --- | --- |
|  |  |  |  | HR (95% C.I.) | P |
| Age at SARS-CoV-2 infection | 10-year effect | 180 | 44 | 1.58 (1.22-2.05) | 0.0006 |
| Year of SARS-CoV-2 infection | 2020-2021 | 74 | 29 | 4.58 (2.42-8.66) | <0.0001 |
|  | 2022 | 106 | 15 | 1.00 |  |
| Time from CAR T-cell infusion to SARS CoV-2 infection | ≤ 3 months | 38 | 15 | 2.27 (1.20-4.27) | 0.01 |
|  | > 3 months | 142 | 29 | 1.00 |  |

Model 1

Model 2

| **Variable** |  | **Patients** | **Deaths** | **Multivariate analysis** | |
| --- | --- | --- | --- | --- | --- |
|  |  |  |  | HR (95% C.I.) | P |
| Age at SARS-CoV-2 infection | 10-year effect | 164 | 40 | 1.71 (1.29-2.27) | 0.0002 |
| Year of SARS-CoV-2 infection | 2020-2021 | 73 | 29 | 5.24 (2.54-10.80) | <0.0001 |
|  | 2022 | 91 | 11 | 1.00 |  |
| Performance status | 10-point effect | 164 | 40 | 0.70 (0.61-0.80) | <0.0001 |

Model 3

| **Variable** |  |  | **Patients** | **Deaths** | **Multivariate analysis** | |
| --- | --- | --- | --- | --- | --- | --- |
|  |  |  |  |  | HR (95% C.I.) | P |
| Age at SARS-CoV-2 infection |  | 10-year effect | 176 | 44 | 1.57 (1.20-2.04) | 0.0009 |
| Year of SARS-CoV-2 infection |  | 2020-2021 | 73 | 29 | 4.13 (2.16-7.91) | <0.0001 |
|  |  | 2022 | 103 | 15 | 1.00 |  |
| Disease status at time of SARS-CoV-2 infection |  | CR | 130 | 19 | 1.00 |  |
|  |  | No CR | 46 | 25 | 4.06 (2.22-7.43) | <0.0001 |
